# One‐year Follow‐up Results of the Optimal Thromboprophylaxis in Elderly Chinese Patients with Atrial Fibrillation (ChiOTEAF) registry

**DOI:** 10.1002/joa3.12608

**Published:** 2021-08-11

**Authors:** Yutao Guo, Hao Wang, Agnieszka Kotalczyk, Yutang Wang, Gregory Y.H. Lip, the ChiOTEAF Registry Investigators

**Affiliations:** ^1^ Department of Pulmonary Vessel and Thrombotic Disease Sixth Medical Centre Chinese PLA General Hospital Beijing China; ^2^ Liverpool Centre for Cardiovascular Science University of Liverpool and Liverpool Heart & Chest Hospital Liverpool UK; ^3^ Department of Cardiology Second Medical Center Chinese PLA General Hospital Beijing China; ^4^ Department of Cardiology Congenital Heart Diseases and Electrotherapy Medical University of Silesia Silesian Centre for Heart Diseases Zabrze Poland; ^5^ Aalborg Thrombosis Research Unit Department of Clinical Medicine Aalborg University Aalborg Denmark

**Keywords:** atrial fibrillation, mortality, prognosis, registry, stroke prevention, thromboprophylaxis

## Abstract

**Background:**

The high prevalence of atrial fibrillation (AF) in the very elderly population (aged >80 years) might be underestimated. The elderly are at increased risk of both fatal stroke and bleeding. The Optimal Thromboprophylaxis in Elderly Chinese Patients with Atrial Fibrillation (ChiOTEAF) registry provides contemporary management strategies among the elderly Chinese patients in the new era of non‐vitamin K antagonists.

**Objective:**

To present the 1‐year follow‐up data from the ChiOTEAF registry, focusing on the use of antithrombotic therapy, rate vs. rhythm control strategies, and determinants of mortality and stroke.

**Methods:**

The ChiOTEAF registry analyzed consecutive AF patients presenting in 44 centers from 20 Chinese provinces from October 2014 to December 2018. Endpoints of interest were mortality, thromboembolism, major bleedings, cardiovascular comorbidities, and hospital re‐admissions.

**Results:**

Of the 7077 patients enrolled at baseline, 657 patients (9.3%) were lost to the follow‐up and 435 deaths (6.8%) occurred. The overall use of anticoagulants remains low, approximately 38% of the entire cohort at follow‐up, with similar proportions of vitamin K antagonists (VKA) and non‐vitamin K antagonists (NOACs). Antiplatelet therapy was used in 38% of the entire cohort at follow‐up, and more commonly among high‐risk patients (41%). Among those on a NOAC at baseline, 22.4% switched to antiplatelet therapy alone after one year.

Independent predictors of stroke/transient ischemic attack/peripheral embolism and/or mortality were age, heart failure, chronic kidney disease, prior ischemic stroke, dementia, and chronic obstructive pulmonary disease.

**Conclusions:**

The ChiOTEAF registry provides contemporary data on AF management, including stroke prevention. The poor adherence of NOACs and common use of antiplatelet in these high‐risk elderly population calls for multiple comorbidities management.

## Graphical abstract

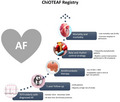

General outcomes of the ChiOTEAF registry. AF, atrial fibrillation.

**Abbreviations**
AF: atrial fibrillation
AFFIRM: The Atrial Fibrillation Follow‐up Investigation of Rhythm Management
CHA2DS2‐VASc: Congestive heart failure or left ventricular dysfunction Hypertension, Age ≥75 (doubled), Diabetes, Stroke (doubled)‐Vascular disease, Age 65‐74, Sex category
CI: confidence interval
ECG: electrocardiography
EORP‐AF: EURObservational Research Programme Atrial Fibrillation
ESC: European Society of Cardiology
HAS‐BLED: hypertension, abnormal renal/ liver function, stroke, bleeding history or predisposition, labile international normalized ratio, elderly, drugs/ alcohol concomitantly
HR: hazard ratio
IQR: inter‐quartile range
mAFA: mobile AF Application
NOAC: non‐vitamin K antagonist
OAC: oral anticoagulant
RCT: randomized control trial
SD: standard deviation
TIA: transient ischemic attack
VKA: vitamin K antagonist: ChiOTEAF: Optimal Thromboprophylaxis in Elderly Chinese Patients with Atrial Fibrillation

## 1 INTRODUCTION

Atrial fibrillation (AF) incidence is increasing over the past decade^1^; patients with AF are older and overburdened with multimorbidity.^2^, ^3^ The prevalence of AF in the elderly (aged >80 years) ranges from 10% to 17%^4^; however, the exact numbers might be underestimated due to asymptomatic AF. Likewise, age is an independent risk factor for adverse outcomes in patients with AF,^5^ and the elderly are at a high risk of fatal ischemic stroke and major bleeding.^6^

Recent data demonstrate the beneficial effect of oral anticoagulants (OACs) for stroke prevention in elderly patients with AF.^7^, ^8^, ^9^, ^10^, ^11^, ^12^ One meta‐analysis showed a significant reduction in the risk of stroke and systemic embolism without increasing major bleeding events among the elderly treated with non‐vitamin K antagonist OACs (NOACs) compared with vitamin K antagonists (VKAs).^13^ A Taiwanese cohort study of extreme elderly (aged >90) AF patients showed superior effectiveness and safety of NOACs compared with VKAs.^11^ Furthermore, the use of NOACs was associated with a reduction in adverse events, especially the risk of intracranial hemorrhage.^11^ Despite the clear benefit of OAC therapy is maintained in elderly patients with AF, “real‐world” data showed that OACs are substantially underused^14^, ^15^, ^16^, ^17^, ^18^ due to a fear of bleeding, especially among those with frailty or dementia.^19^ Of note, the NOACs showed better efficacy and safety among Asian patients compared with non‐Asians.^20^

The introduction of the NOACs has led to a major change in the landscape of stroke prevention in AF, but limited contemporary nationwide data are evident from China. Thus, the prospective, nationwide Optimal Thromboprophylaxis in Elderly Chinese Patients with Atrial Fibrillation (ChiOTEAF) registry aimed to explore contemporary regional management strategies, including antithrombotic therapy among the high‐risk AF population of the elderly Chinese patients, in the new era of the NOACs. In this analysis, we present the 1‐year follow‐up data from the ChiOTEAF registry, focusing on the use of antithrombotic therapy, rate vs. rhythm control strategies, and determinants of mortality and stroke.

## 2 METHODS

The protocol of the ChiOTEAF registry has previously been published.^21^ The study was approved by the Central Medical Ethics Committee of Chinese PLA General Hospital, Beijing, China (approval no S2014‐065‐01) and local institutional review boards.

The registry was conducted between October 2014 and December 2018. Briefly, the registry population comprises consecutive in‐ and outpatients presenting with AF to cardiologists (mainly), neurologist, and surgeons, enrolled in 44 sites from 20 Chinese provinces. The main inclusions criteria were age ≥65 years (for the extended analysis, AF patients aged >50 years were included) and the qualifying AF event in the 12 months prior to enrolment (recorded by a 12‐lead ECG or 24 hours ECG Holter).

Data were collected at the moment of enrolment and during the follow‐up visits (including patient visit and/or chart review and/or telephone follow‐up) by any investigator and reported into an electronic case report form. Follow‐up was performed by the local investigators, initially at 6 and 12 months in the first year and annually for the next 2 years. Endpoints of interest were mortality, thromboembolism, major bleedings, cardiovascular comorbidities, and hospital re‐admissions. For this analysis, we focused on 1‐year outcomes.

The ChiOTEAF registry has common definitions and protocol for the EURObservational Research Programme Atrial Fibrillation (EORP‐AF) General Registry.^22^ Based on the ESC guidelines,^23^ thromboembolic risk was categorized using the CHA_2_DS_2_‐VASc score.^5^ “Low‐risk” patients were defined as males with a CHA_2_DS_2_‐VASc 0 or females with a CHA_2_DS_2_‐VASc 1; “moderate risk” was defined as male patients with a CHA_2_DS_2_‐VASc score 1 or females with a CHA_2_DS_2_‐VASc 2; and “high risk” was defined as CHA_2_DS_2_‐VASc score ≥2. Bleeding risk was assessed based on the HAS‐BLED bleeding score.^23^

### 2.1 Statistical analyses

Univariate analysis was applied to continuous and categorical variables. Continuous variables were reported as mean+SD and/or as median and inter‐quartile range (IQR). Among‐group comparisons were made using a non‐parametric test (Kruskal–Wallis test). Categorical variables were reported as percentages, and the χ^2^ test or Fisher's exact test (if required) was used for among‐group comparisons.

All the statistically significant variables at univariate analysis and variables considered of relevant clinical interests were included in the multivariable model to distinguish the independent predictors of all‐cause death and/or stroke/transient ischemic attack (TIA)/peripheral embolism during the 1‐year follow‐up period. A Cox proportional hazard model was performed by adjusting for the following covariates: sex, hypertension, coronary artery disease, liver dysfunction, and prior major bleeding. All Cox regression analyses were reported as hazard ratio (HR) and 95% confidence interval [CI]. A two‐sided *P*‐value of <.05 was considered statistically significant.

## 3 RESULTS

**FIGURE 1:**
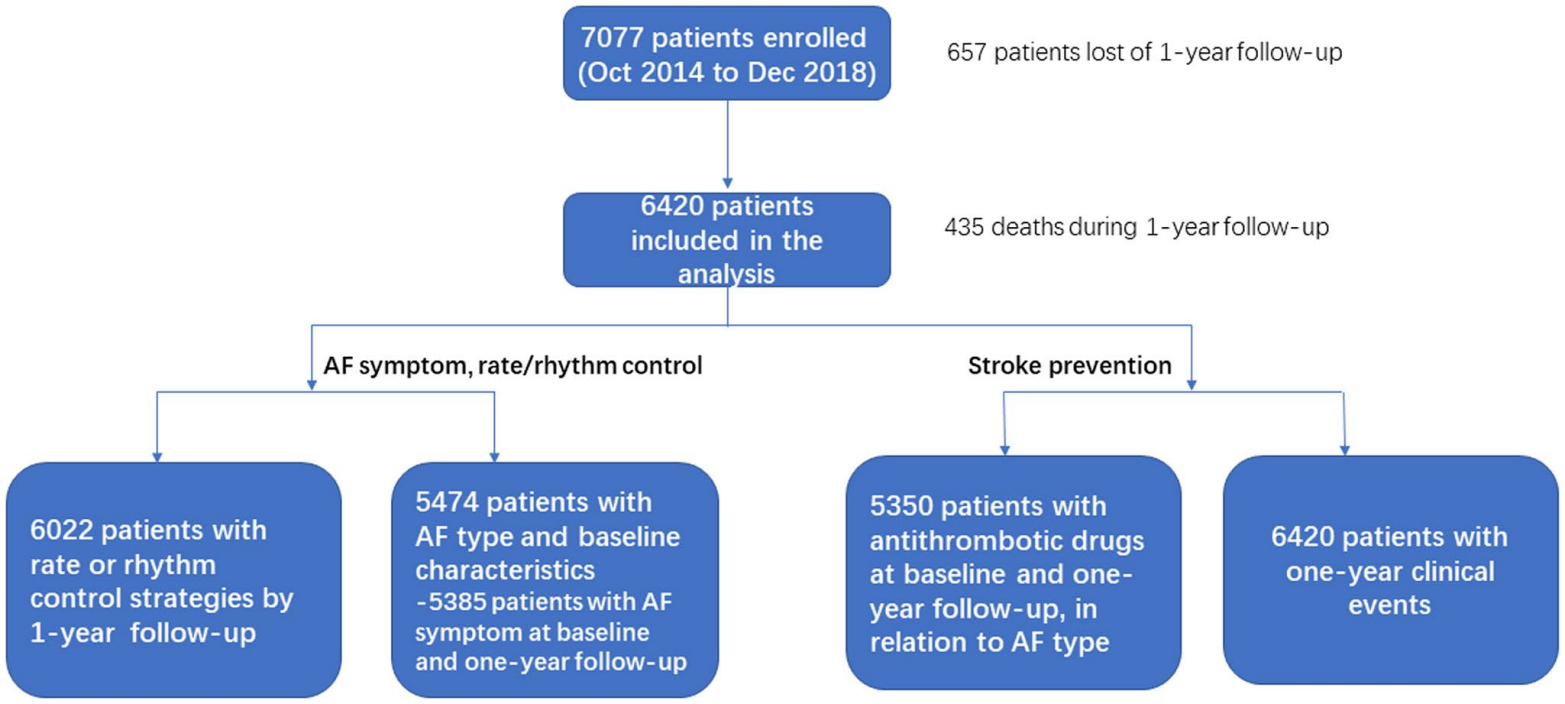
Patient flow as part of the ChiOTEAF registry. AF, atrial fibrillation

Available data on patient demography and baseline characteristics in relation to clinical AF subtype are summarized in Table 1, and the patient disposition is shown in Figure 1. Of the 7077 patients enrolled at baseline, 657 patients (9.3%) were lost to follow‐up and 435 deaths (6.8%) occurred (Figure 1).

**TABLE 1:** Patient demography in relation to clinical subtype of atrial fibrillation

	Total (n = 5474)	First detected (n = 886)	Paroxysmal (n = 2403)	Persistent (n = 980)	Long‐standing persistent AF (n = 181)	Permanent (n = 838)	Unknown (n = 185)	*P*‐value
Age (years) [mean ± SD]	73.4 ± 10.6	74.8 ± 10.5	71.7 ± 10.6	73.6 ± 10.6	74.9 ± 11.1	76.8 ± 9.4	70.7 ± 9.9	<0.001
Age (years) [Median (IQR)]	75.0 (65.0‐82.0)	77.0 67.7‐83.0)	72.0 64.0‐80.0)	75.0 (65.0‐81.0)	76.0 (66.0‐83.5)	79.0 (70.0‐84.0)	70.0 (63.0‐78.5)	<0.001
Age (years)								
≤65, n (%)	1396 (25.5%)	186 (21.0%)	738 (30.7%)	245 (25.0%)	43 (23.8%)	122 (14.6%)	62 (33.5%)	<0.001
>65, n (%)	4076 (74.5%)	700 (79.0%)	1665 (69.3%)	734 (75.0%)	138(76.2%)	716 (85.4%)	123 (66.5%)	<0.001
Gender								
Male, n (%)	3300 (60.3%)	517 (58.4%)	1437 (59.8%)	610 (62.2%)	111 (61.3%)	513 (61.2%)	112 (60.2%)	0.612
Female, n (%)	2174 (39.7%)	369 (41.6%)	966 (40.2%)	370 (37.8%)	70 (38.7%)	325 (38.8%)	74 (39.8%)	
Stroke risk based on CHA_2_DS_2_‐VASc score								<0.001
Low risk, n (%)	312 (5.7%)	39 (4.4%)	186 (7.7%)	38 (3.9%)	5 (2.8%)	24 (2.9%)	20 9(10.8%)	
Moderate risk, n (%)	628 (11.5%)	96 (10.8%)	328 (13.6%)	111 (11.3%)	15 (8.3%)	51 (6.1%)	27 (14.5%)	
High risk, n (%)	4534 (82.8%)	751 (84.8%)	1889 (78.6%)	831 (84.8%)	161 (89.0%)	763 (91.1%)	139 (74.7%)	
HAS‐BLED score class								
0‐2, n (%)	4326 (79.0%)	697 (78.7%)	1974 (82.1%)	760 (77.6%)	133 (73.5%)	593 (70.8%)	169 (90.9%)	<0.001
≥ 3, n (%)	1148 (21.0%)	189 (21.3%)	429 (17.9%)	220 (22.4%)	48 (26.5%)	245 (29.2%)	17 (9.1%)	
Comorbidities								
Hypertension, n (%)	3426 (62.6%)	574 (64.8%)	1445 (60.1%)	635 (64.8%)	114 (63%)	574 (68.4%)	84 (45.4%)	<0.001
Coronary artery disease, n (%)	2453 (44.8%)	416 (46.9%)	1082 (45%)	405 (41.3%)	104 (57.5%)	377 (45%)	69 (37.3%)	<0.001
Heart failure, n (%)	1758 (32.1%)	310 (3.5%)	523 (21.8%)	366 (37.3%)	108 (59.7%)	391 (46.7%)	60 (32.4%)	<0.001
Diabetes mellitus, n (%)	1415 (25.8%)	215 (24.3%)	615 (25.6%)	248 (25.3%)	57 (31.5%)	250 (29.8%)	30 (16.2%)	0.008
Prior ischemic stroke, n (%)	1100 (20.1%)	152 (17.2%)	443 (18.4%)	200 (20.4%)	38 (21.0%)	247 (29.5%)	20 (10.8%)	<0.001
Chronic kidney disease, n (%)	587 (10.7%)	105 (11.9%)	206 (8.6%)	114 (11.6%)	23 (12.7%)	129 (15.4%)	10 (5.4%)	<0.001
Liver disease, n (%)	199 (3.6%)	35 (3.9%)	84 (3.5%)	10 (1%)	11 (6%)	20 (2.4%)	9 (4.9%)	0.098
Chronic obstructive pulmonary disease, n (%)	373 (6.8%)	70 (7.9%)	118 (4.9%)	66 (6.7%)	11 (6%)	99 (11.8%)	9 (4.9%)	<0.001
Dementia, n (%)	115 (2.1%)	26 (2.9%)	42 (1.7%)	16 (1.6%)	7 (3.9%)	24 (2.9%)	0 (0%)	0.007
Moderate/severe mitral stenosis	153 (2.8%)	19 (2.1%)	47 (1.9%)	27 (2.7%)	13 (7.2%)	46 (5.5%)	1 (0.5%)	<0.001
Current symptoms at 1‐year follow‐up, n (%)	1098 (20.4%)	160 (18.4%)	456 (19.1%)	242 (25.1%)	50 (27.6%)	154 (19%)	37 (19.8%)	<0.001
Palpitations, n (%)	859 (15.9%)	141 (16.3%)	366 (15.4%)	186 (19.3%)	33 (18.6%)	104 (12.9%)	29 (15.4%)	<0.001
Dizziness, n (%)	19 (0.4%)	3 (0.3%)	4 (0.2%)	4 (0.4%)	0 (0)	5 (0.6%)	3 (1.6%)	0.018
General non‐wellbeing, n (%)	98 (1.8%)	35 (4.0%)	19 (0.8%)	21 (2.2%)	7 (4.0%)	14 (1.7%)	2 (1.1%)	<0.001
Fatigue, n (%)	25 (0.5%)	5 (0.6%)	5 (0.2%)	2 (0.2%)	2 (1.1%)	11 (1.4%)	0 (0)	<0.001
Shortness of breath, n (%)	129 (2.4%)	17 (2.0%)	32 (1.3%)	30 (3.1%)	9 (5.1%)	35 (4.3%)	6 (3.2%)	<0.001
Chest pain, n (%)	45 (0.8%)	8 (0.9%)	12 (0.5%)	13 (1.3%)	0 (0)	8 (1.0%)	4 (2.1%)	<0.001
Fear/anxiety, n (%)	105 (1.9%)	38 (4.4%)	16 (0.7%)	19 (2.0%)	8 (4.5%)	15 (1.9%)	9 (4.8%)	<0.001
Other, n (%)	18 (0.3%)	3 (0.3%)	2 (0.1%)	5 (0.5%)	3 (1.7%)	4 (0.5%)	1 (0.5%)	0.006

Abbreviations: CHA_2_DS_2_‐VASc, Congestive heart failure or left ventricular dysfunction Hypertension, Age ≥75 (doubled), Diabetes, Stroke (doubled)‐Vascular disease, Age 65‐74, Sex category; HAS‐BLED, hypertension, abnormal renal/ liver function, stroke, bleeding history or predisposition, labile international normalized ratio, elderly, drugs/ alcohol concomitantly; IQR, inter‐quartile range;SD, standard deviation.

The median age of AF patients (n = 5474, 39.7% female) in relation to clinical subtype was 75.0 (65.0‐82.0) years, with a vast majority of patients at high risk of stroke (CHA_2_DS_2_‐VASc score ≥2) (Table 1). Analysis of AF subtypes showed that those patients with permanent AF were older, but no statistically significant difference was found in a gender ratio between groups. Differences in the risk of stroke and bleeding (HAS‐BLED score ≥3) strata were evident, with more high‐risk patients in the subgroups of permanent and long‐standing persistent AF (Table 1). Patients were overburdened with multi‐morbidity (particularly patients with long‐standing persistent and permanent AF), including hypertension (62.6%), coronary artery disease (44.8%), heart failure (32.1%), diabetes mellitus (25.8%), prior ischemic stroke (20.1%) chronic kidney disease (10.7%), chronic obstructive pulmonary disease (6.8%), and dementia (2.1%).

### 3.1 Symptoms at follow‐up

Of those patients with reported data, 1098 (20.4%) were symptomatic at 1‐year follow‐up (Table 1), most frequently among persistent and long‐standing persistent AF patients (25.1% and 27.6%, respectively). The most common symptoms at follow‐up were palpitations (15.9%), shortness of breath (2.4%), and fear/anxiety (1.9%).

### 3.2 Antithrombotic therapy

**FIGURE 2:**
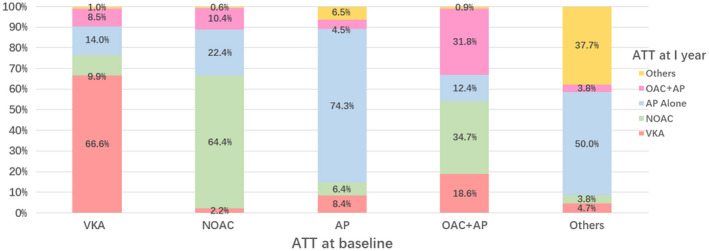
Antithrombotic therapy use at 1 year based on initial/baseline antithrombotic regimen. ATT, antithrombotic therapy; VKA, vitamin K antagonist; NOAC, non‐vitamin K anatagonist; AP, antiplatelet therapy (most commonly aspirin); OAC, oral anticoagulant therapy

Overall, 5350 patients had available data on antithrombotic drugs at baseline and 1‐year follow‐up, in relation to AF type. The use of antithrombotic therapy at a 1‐year follow‐up visit, concerning antithrombotic therapy used at the baseline visit is shown in Figure 2. Of those on a vitamin K antagonist (VKA), 75.1% remained on a VKA, and 9.9% had switched to a NOAC during the follow‐up. Among those on a NOAC at baseline, 2.2% had changed to a VKA and 22.4% to antiplatelet therapy alone. Of those on antiplatelet therapy at the baseline, 14.8% had switched to OAC, and 4.5% had dual therapy (OAC and antiplatelet).

**FIGURE 3:**
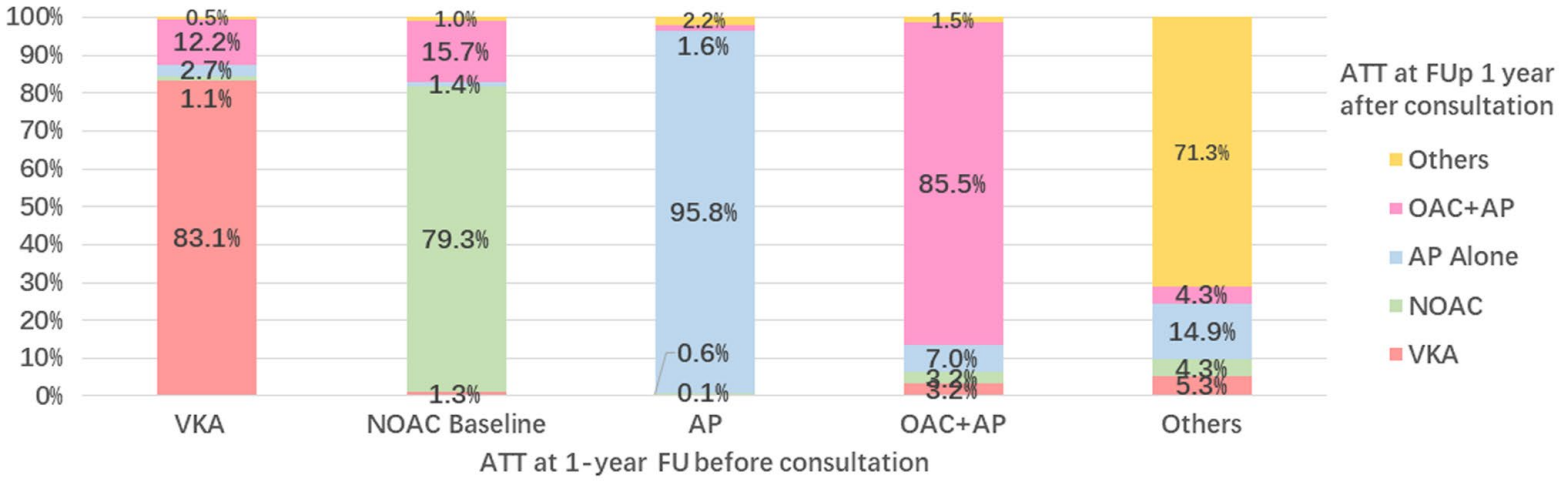
Antithrombotic therapy at 1 year comparing before vs. after visit/consultation. ATT, antithrombotic therapy; FU: follow up. VKA, vitamin K antagonist; NOAC, non‐vitamin K antagonist; AP, antiplatelet therapy (most commonly aspirin); OAC, oral anticoagulant therapy

Drug therapies prescribed at follow‐up are shown in Table 2, summarizing drugs used before (and after) the follow‐up consultation. The overall use of OACs remained low among 5350 AF patients, approximately 37.8%–38.8% of the entire cohort at follow‐up, with similar proportions of a VKA (18.0%–17.8%) and NOACs (20.1%–21.1%) pre and post the follow‐up consultation visit (Table 2, Figure 3). The use of OACs was the highest among persistent and long‐standing persistent AF patients (51.3%, 51.1%, respectively), with significantly lower intake in the subgroup of first detected and paroxysmal AF (33.4%, 34.6%, respectively). The NOACs were more common among long‐persistent AF (30.5%), while they were used only in 14.7% of those with persistent AF. Antiplatelet therapy was used in 37.9%–38.1% of the entire cohort at follow‐up and more commonly among first detected AF (44.0%–44.2%).

**TABLE 2:** Drug therapies prescribed at follow‐up

	Total (n = 5350)	First detected (n = 859)	Paroxysmal (n = 2370)	Persistent (n = 958)	Long‐standing persistent AF (n = 174)	Permanent (n = 808)	Unknown (n = 181)	*P*‐value
(a) Antithrombotic drugs by AF subgroup								
Oral anticoagulation drug								
Pre‐follow‐up consultation, n (%)	2021 (37.8%)	272 (31.7%)	813 (34.3%)	486 (51.3%)	79 (45.4%)	331 (40.9%)	40 (22.1%)	<0.001
After follow‐up consultation, n (%)	2074 (38.8%)	287 (33.4%)	821 (34.6%)	486 (51.3%)	89 (51.1%)	340 (42.1%)	51 (28.2%)	<0.001
VKA								
Pre‐follow‐up consultation, n (%)	964 (18.0%)	121 (14.1%)	321 (13.5%)	236 (24.6%)	37 (21.3%)	222 (27.5%)	27 (14.9%)	<0.001
After follow‐up consultation, n (%)	950 (17.8%)	122 (14.2%)	308 (13.0%)	230 (24.0%)	37 (21.3%)	221 (27.4%)	32 (17.7%)	<0.001
NOAC								
Pre‐follow‐up consultation, n (%)	1074 (20.1%)	152 (17.7%)	500 (21.1%)	253 (26.4%)	42 (24.1%)	113 (14.0%)	14 (7.7%)	<0.001
After follow‐up consultation, n (%)	1129 (21.1%)	165 (19.2%)	515 (21.7%)	258 (26.9%)	53 (30.5%)	119 (14.7%)	19 (10.5%)	<0.001
Antiplatelet drug								
Pre‐follow‐up consultation, n (%)	2037 (38.1%)	378 (44.0%)	918 (38.7%)	310 (32.4%)	61 (35.1%)	302 (37.4%)	68 (37.6%)	<0.001
After follow‐up consultation, n (%)	2029 (37.9%)	380 (44.2%)	907 (38.3%)	310 (32.4%)	61 (35.1%)	305 (37.7%)	66 (36.5%)	<0.001

	Total (n = 5350)	Low (n = 376)	Moderate (n = 714)	High (n = 4260)	*P*‐value
(b) Antithrombotic therapy by stroke risk strata					
Oral anticoagulation drug					
Pre‐follow‐up consultation, n (%)	2021 (37.8%)	105 (27.9%)	244 (34.2%)	1672 (39.2%)	<0.001
After follow‐up consultation, n (%)	2074 (38.8%)	109 (29.0%)	256 (35.8%)	1709 (40.1%)	<0.001
VKA					
Pre‐follow‐up consultation, n (%)	964 (18.0%)	50 (13.3%)	116 (16.2%)	798 (18.7%)	0.013
After follow‐up consultation, n (%)	950 (17.8%)	48 (12.8%)	117 (16.4%)	785 (18.4%)	0.013
NOAC					
Pre‐follow‐up consultation, n (%)	1074 (20.1%)	56 (14.9%)	129 (18.1%)	889 (20.9%)	0.008
After follow‐up consultation, n (%)	1129 (21.1%)	61 (16.2%)	139 (19.5%)	929 (21.8%)	0.02
Antiplatelet drug					
Pre‐follow‐up consultation, n (%)	2037 (38.1%)	89 (23.7%)	189 (26.4%)	1759 (41.3%)	<0.001
After follow‐up consultation, n (%)	2029 (37.9%)	89 (23.7%)	186 (26.1%)	1754 (41.2%)	<0.001

	Total (n = 5350)	First detected (n = 859)	Paroxysmal (n = 2370)	Persistent (n = 958)	Long‐standing persistent AF (n = 174)	Permanent (n = 808)	Unknown (n = 181)	P‐value
(c) Rhythm/rate control drugs (at follow‐up after consultation)								
Class Ic (propafenone), n (%)	206 (3.9%)	19 (2.2%)	139 (5.9%)	37 (3.9%)	2 (1.1%)	5 (0.6%)	4 (2.2%)	<0.001
Beta‐blockers, n (%)	2918 (54.5%)	466 (54.2%)	1241 (52.4%)	588 (61.4%)	120 (69.0%)	413 (51.1%)	90 (49.7%)	<0.001
Class III (amiodarone), n (%)	465 (8.7%)	62 (7.2%)	301 (12.7%)	70 (7.3%)	15 (8.6%)	10 (1.2%)	7 (3.9%)	<0.001
Digitalis (mainly digoxin), n (%)	547 (10.2%)	79 (9.2%)	137 (5.8%)	120 (12.5%)	29 (16.7%)	155 (19.2%)	27 (14.9%)	<0.001

Abbreviations: AF, atrial fibrillation; NOAC, non‐vitamin K antagonist; VKA, vitamin K antagonist.

Table 2B shows the use of antithrombotic therapy by stroke risk strata (based on the CHA_2_DS_2_‐VASc score). OACs were used in 27.9%–29.0% of low‐risk patients, in 34.2%–35.8% of moderate‐risk, and in 39.2%–40.1% of high‐risk patients, while the NOACs were used in 14.9%–16.2% of low, 18.1%–19.5% of moderate and 20.9%–21.8% of high‐risk patients, respectively. Antiplatelet therapy was used in 41.2%–41.3% of patients at high risk of stroke.

### 3.3 Rate and rhythm control strategy

For the analysis of rate and rhythm control strategies, 6022 patients with available data by 1‐year follow‐up were included. Drugs used for rhythm and rate control therapy at follow‐up are summarized in Table 2C. Beta‐blockers (54.5%) and digitalis (10.2%) remained the most common drugs used, especially in persistent and long‐standing persistent AF; while Class Ic and III drugs were more often used in paroxysmal AF (5.9% and 12.7%, respectively).

Among patients managed with rate control at baseline, only 4.2% continued a rate control strategy, while rhythm control was considered in 43.1% (Figure S1). Of those considered for a rhythm control at baseline, 23.9% continued the strategy, and 16.4% were eventually considered for a rate control therapy.

Table 3 shows the interventions performed by the 1‐year follow‐up. Any rhythm control intervention was performed in 9% of the overall cohort—especially among persistent and long‐standing persistent AF patients (12.8 and 17.6%, respectively). Catheter ablation was performed in 5.5% of the population, commonly among paroxysmal AF patients (8%); whereas pacemaker implantation was required in 6.9% of permanent AF patients.

**TABLE 3:** Interventions performed by 1‐year follow‐up

	Total (n = 6022)	First detected (n = 125)	Paroxysmal (n = 3778)	Persistent (n = 802)	Long‐standing persistent AF (n = 210)	Permanent (n = 925)	Unknown (n = 182)	P‐value
Rhythm control intervention, n (%)	545 (9.05%)	12 (9.60%)	303 (8.02%)	103 (12.84%)	37 (17.62%)	81 (8.76%)	9 (4.95%)	<0.001
Pharmacological cardioversion, n (%)	140 (2.32%)	3 (2.40%)	107 (2.83%)	16 (2.00%)	9 (4.29%)	4 (0.43%)	1 (0.55%)	<0.001
Electrical cardioversion, n (%)	21 (0.35%)	2 (1.60%)	8 (0.21%)	7 (0.87%)	0 (0)	2 (0.22%)	2 (1.10%)	0.003
Catheter ablation, n (%)	332 (5.51%)	4 (3.20%)	303 (8.02%)	13 (1.62%)	1 (0.48%)	3 (0.32%)	8 (4.40%)	<0.001
Pacemaker implantation, n (%)	329 (5.46%)	4 (3.20%)	206 (5.45%)	40 (4.99%)	14 (6.67%)	64 (6.92%)	1 (0.55%)	0.013
Implantable defibrillator, n (%)	26 (0.43%)	1 (0.80%)	16 (0.42%)	3 (0.37%)	1 (0.48%)	5 (0.54%)	0 (0)	0.911
AF surgery, n (%)	54 (0.90%)	1 (0.81%)	34 (0.91%)	6 (0.75%)	1 (0.48%)	2 (0.22%)	10 (5.81%)	<0.001

Abbreviation: AF, atrial fibrillation.

### 3.4 Mortality and morbidity

After one year, 6.8% (435/6420) of the patients enrolled in the study died between the enrolment and the 1‐year follow‐up visit (Table 4). Causes were categorized as cardiovascular (28.5%; 124/435) and non‐cardiovascular (56%; 244/435).

**TABLE 4:** Mortality and morbidity during the 1‐year follow‐up

	Total (n = 6420)	First detected (n = 948)	Paroxysmal (n = 2461)	Persistent (n = 1017)	Long‐standing persistent AF (n = 188)	Permanent (n = 887)	Unknown/missing data (n = 919)
(a) Mortality							
Death, n (%)	435 (6.8%)	84 (8.9%)	83 (3.4%)	55 (5.4%)	16 (8.5%)	88 (9.9%)	109 (11.9%)
Causes of death:							
Cardiovascular, n (%)	124 (28.5%)	36 (42.9%)	18 (21.7%)	15 (27.3%)	3 (18.8%)	25 (28.4%)	28 (25.7%)
Non‐cardiovascular, n (%)	244 (56.1%)	40 (47.6%)	55 (66.3%)	31 (56.3%)	8 (50%)	52 (59.1%)	58 (53.2%)
Unknown	67 (15.4%)	8 (9.5%)	10 (12%)	9 (16.4)	5 (31.2)	11 (12.5%)	23 (21.1%)
(b) Morbidities							
ACS, n (%)	67 (1%)	15 (1.6%)	9 (0.4%)	10 (1%)	4 (2.1%)	12 (1.4%)	17 (1.8%)
Heart failure, n (%)	108 (1.7%)	22 (2.3%)	19 (0.8%)	11 (1%)	5 (2.7%)	16 (1.8%)	35 (3.80)
Any thromboembolic event	102 (1.6%)	13 (1.4%)	17 (0.7%)	16 (1.6%)	5 (2.7%)	24 (2.7%)	27 (2.9%)
Ischemic stroke, n (%)	62 (1%)	8 (0.8%)	13 (0.5%)	5 (0.5%)	4 (2.1%)	17 (1.9%)	15 (1.6%)
TIA, n (%)	9 (0.1%)	3 (0.3%)	0 (0%)	0 (0%)	1 (0.5%)	4 (0.5%)	1 (0.1%)
Peripheral/pulmonary embolism, n (%)	18 (0.2%)	3 (0.3%)	0 (0%)	2 (0.2%)	0 (0%)	4 (0.5%)	9 (1%)
Intracranial hemorrhage, n (%)	18 (0.2%)	0 (0%)	3 (0.1%)	5 (0.5%)	0 (0%)	8 (1%)	2 (0.2%)
Extracranial bleeding, n (%)	84 (1.3%)	16 (1.7%)	18 (0.7%)	8 (0.8%)	6 (3.2%)	15 (1.7%)	21 (2.3%)
Readmissions for arrhythmias, n (%)	146 (2.3%)	26 (2.7%)	37 (1.5%)	23 (2.3%)	12 (6.4%)	20 (2.3%)	28 (3%)
Recurrent AF/atrial flutter, n (%)	47 (0.7%)	11 (1.2%)	13 (0.5%)	2 (0.2%)	0 (0%)	5 (0.6%)	16 (1.7%)

Abbreviations: ACS, acute coronary syndrome; AF, atrial fibrillation; TIA, transient ischemic attack.

During the 1‐year follow‐up, there were 321 cardiac re‐admissions reported; 146 for arrhythmias (47 for AF/atrial flutter recurrence), 67 for acute coronary syndrome, and 108 for heart failure. In this high‐risk cohort, 102 thromboembolism complications (including 62 ischemic strokes, 9 TIAs, and 18 systemic embolisms) and 102 major bleedings (including 18 intracranial hemorrhages) occurred. Patients with long‐standing persistent and permanent AF were at the highest risk of both ischemic stroke/TIA and bleeding.

### 3.5 Multivariate analysis

A Cox proportional hazard model was compiled to establish clinical factors associated with the composite outcome of stroke/TIA/peripheral embolism and/or death (Table 5). For stroke/TIA/peripheral embolism and/or mortality, independent predictors were age (HR: 3.75; 95% CI: 2.85‐4.94; *P* <.001), heart failure (HR: 1.93; 95% CI: 1.58‐2.34; *P* <.001), chronic kidney disease (HR: 1.82; 95% CI: 1.48‐2.25; *P* <.001), prior ischemic stroke (HR: 1.28; 95% CI: 1.05‐1.56; *P* =.015), dementia (HR: 2.40; 95% CI: 1.84‐3.14; *P* <.001) and chronic obstructive pulmonary disease (HR: 1.72; 95% CI: 1.38‐2.14; *P* <.001).

**TABLE 5:** Multivariate analysis

Clinical variable	Hazard ratio	95% CI		*P*‐value
		Low	High	
(a) Stroke/TIA/peripheral embolism and/or mortality				
Age >75 years	3.75	2.85	4.94	<0.001
Heart failure	1.93	1.58	2.34	<0.001
Chronic kidney disease	1.82	1.48	2.25	<0.001
Prior ischemic stroke	1.28	1.05	1.56	0.015
Dementia	2.40	1.84	3.14	<0.001
COPD	1.72	1.38	2.14	<0.001
(b) Mortality				
Age >75 years	4.02	2.95	5.49	<0.001
Heart failure	2.24	1.78	2.81	<0.001
Chronic kidney disease	1.98	1.59	2.48	<0.001
Prior ischemic stroke	1.21	0.97	1.49	0.09
Dementia	2.41	1.80	3.21	<0.001
COPD	1.59	1.26	2.03	<0.001
(c) Stroke/TIA/peripheral embolism				
Age >75 years	2.66	1.42	5.00	0.002
Heart failure	1.11	0.66	1.87	0.696
Chronic kidney disease	1.00	0.52	1.91	0.997
Prior ischemic stroke	1.90	1.15	3.15	0.012
Dementia	1.90	0.86	4.20	0.11
COPD	2.85	1.60	5.07	<0.001

Abbreviations: CI, confidence interval; COPD, chronic obstructive pulmonary disease; TIA—transient ischemic attack. Adjusted for sex, diabetes mellitus, hypertension, coronary artery disease, liver dysfunction, prior major bleeding.

## 4 DISCUSSION

In this 1‐year follow‐up analysis of high‐risk elderly patients with AF, our principal findings are as follows: (a) patients are frequently asymptomatic, but in a fifth of AF patients, symptoms are present (mostly palpitations, shortness of breath, and fear/anxiety); (b) the use of OAC remained low, less than 40% of patients, with similar proportions of VKA and NOACs; (c) rhythm control was infrequent, with any rhythm control intervention being performed in 9% of patients (and catheter ablation in only 5.5%); (d) 1‐year mortality was high (6.8%, with the majority being non‐cardiovascular deaths) and independent predictors of mortality were age, heart failure, chronic kidney disease, chronic obstructive pulmonary disease and dementia; and (e) hospital re‐admissions were common, especially for arrhythmic causes.

The ChiOTEAF registry is the first contemporary nationwide prospective survey focused on management practices among Chinese cardiologists, with associated follow‐up data, since the introduction of NOACs. It was designed to have aligned definitions of clinical outcomes and a common protocol with the EORP‐AF registry to compare AF management between European and Chinese populations.

While patients are frequently asymptomatic, symptoms at 1‐year follow‐up are common among persistent and long‐term persistent AF patients (but not paroxysmal AF), particularly palpitations and shortness of breath. Of note, over 50% of patients were treated with beta‐blockers; and rhythm control drugs were used in 12.6% of patients (particularly amiodarone in the paroxysmal AF subgroup). Consistent with symptom‐based management, any rhythm control intervention was limited and performed only in 9% of the overall cohort (most commonly in persistent and long‐term persistent AF).

Given that many elderly AF patients are asymptomatic, opportunistic screening is recommended for early AF detection in those aged ≥65 years.^24^ Likewise, the rhythm control strategy should be recommended for symptomatic patients to mitigate their symptoms and improve the quality of life.^24^ In the elderly, rate control is often the management of choice^25^; while rhythm control may be a preferable strategy among younger AF patients (aged <65 years), resulting in a higher rate of sinus rhythm restoration and a lower risk of all‐cause mortality than rate control strategy.^26^ An increasingly common approach is to use catheter ablation as first‐line treatment to reduce AF‐related adverse clinical outcomes among patients with recently diagnosed AF, with superior results compared to anti‐arrhythmic drugs.^27^, ^28^, ^29^

The ChiOTEAF registry showed that the overall use of OACs was relatively low (38% of patients at follow‐up), with similar uptake of a VKA (18%) and NOACs (20%–21%) among Chinese elderly. In comparison, data from European registries shows over 80% of AF patients being anticoagulated, and NOACs account for 40% of OACs.^22^, ^30^, ^31^, ^32^ However, an improvement in the use of OACs among Chinese patients can be observed as compared to the data from the Clinical Epidemiology of Atrial Fibrillation in Asia and previous Chinese registries.^33^, ^34^ The use of OACs is increasing steadily, and most recently, the Chinese Atrial Fibrillation Registry Study showed that 36.5% of patients with AF and CHA_2_DS_2_‐VASc scores ≥2 were anticoagulated.^34^ Indeed, prior papers^33^, ^34^ have highlighted the poor uptake of OACs in China, and the reasons may be multifactorial. These include patient's perceptions, physician/prescriber concerns about bleeding and costs (in the case of NOACs).

Despite guideline recommendations, we found that antiplatelet therapy (commonly aspirin) was still used in 23.7% of low‐risk and 41% of high‐risk patients. When a NOAC was discontinued, over a fifth of patients was started on antiplatelet therapy. However, the reasons for this antiplatelet “overuse” in Chinese patients are not evidence‐based; indeed, OACs were found to have superior efficacy with similar safety than aspirin among the elderly with AF.^8^, ^35^ In the EORP‐AF Long‐Term Registry, antiplatelet therapy was prescribed in 20% of patients, while 6.4% had no antithrombotic treatment.^32^ The poor adherence of OACs at 1‐year follow‐up and common use of antiplatelet in ChiOTEAF registry reflected the real‐world clinical practice, partly contributed by patient's risk profile, with complex comorbidities in these elderly population, thus highlighting cardiovascular risk and comorbidities management. Given that antiplatelet therapy is still commonly used in China,^36^ planned analyses of the ChiOTEAF registry might help addressing this “gap” and determine the reasons for OACs withholding in Chinese patients.

Given that stroke prevention is central to AF management, better education and awareness are needed to improve outcomes in this AF population.^37^ Indeed, guideline‐driven anticoagulation is related to significantly better outcomes in the elderly (including lower risk of all‐cause and cardiovascular deaths).^38^ In contrast, both under‐treatment and over‐treatment increases the risk of death and thromboembolism among AF patients (HR: 1.679; 95% CI: 1.202‐2.347 and HR: 1.622; 95% CI: 1.173‐2.23; respectively).^39^ Another study showed that multimorbidity was an independent factor of withholding OAC, while frequent falls and frailty were the most common reasons for non‐prescription of OACs in the elderly.^40^

Furthermore, our data show high morbidity and mortality rates. Indeed, 1‐year mortality was 6.8% in all cohort, particularly from non‐cardiovascular causes. During the follow‐up, 89 thromboembolism complications occurred. Independent predictors for stroke/TIA/peripheral embolism and/or mortality included age, heart failure, chronic kidney disease, prior ischemic stroke, dementia, and chronic obstructive pulmonary disease. Similarly, the European data of AF patients showed that overall mortality rates remained high (5%) during the 2‐year follow‐up, but mostly due to cardiovascular causes (61.8%).^41^ Accordingly in the AFFIRM trial, the diagnosis of heart failure, chronic obstructive pulmonary disease, and osteoporosis were associated with an increased risk of all‐cause mortality among elderly AF patients.^3^

Consistent with other registries, hospital re‐admissions were common in our cohort, especially for cardiac causes (atrial arrhythmias and heart failure). The increasing number of AF‐related hospitalizations is acknowledged as a major healthcare costs.^42^ A recent RCT^43^ assessed the impact of integrated care supported by the mobile AF Application (mAFA) on clinical outcomes among Chinese patients with AF and ≥2 stroke risk factors during a 12‐month follow‐up. The composite endpoint of ischemic stroke/systemic thromboembolism, death, and re‐hospitalization was lower in the mAFA patients than usual care.^43^, ^44^ Among the mAFA group, lower rates of OAC‐related bleeding (due to the mitigation of modifiable bleeding risk factors) and an increase in the use of OAC (from 63.4% to 70.2%) was observed as compared to standard care.^45^ Indeed, implementing digital healthcare models into holistic care pathways of patients with AF may improve patients awareness and treatment acceptance, resulting in better outcomes and OACs compliance.^46^, ^47^, ^48^

### 4.1 Limitations

The primary limitation of the study is its observational nature, and given its modest size, it was not powered to detect differences in some endpoints. Patients were enrolled in 44 centers, which implies a potential variability in the therapeutic strategies for AF. Moreover, the enrolment period was relatively long, which may affect the generalizability of the results. There was a moderate proportion of patients lost to follow‐up (9.3%) consistent with large European registries.^49^ Also, the causes of 67 deaths (15.4%) are unknown, and 919 patients have unknown (182 patients)/missing data (737 patients) of the AF type. Finally, data on anticoagulation control are not currently available for this cohort and cannot be considered in this analysis.

## 5 CONCLUSIONS

The Optimal Thromboprophylaxis in Elderly Chinese Patients with Atrial Fibrillation registry provides contemporary data on AF management, including stroke prevention. The rate of OAC use was <40%, and antiplatelet therapy is still commonly prescribed among high‐risk patients. Given that Chinese patients with AF are increasingly elderly and overburdened with multimorbidity, our large cohort data may help establish best practices to reduce morbidity and mortality.

### 5.1 Clinical perspectives

**S**troke prevention is central to AF management; better education and awareness are needed to improve outcomes in high‐risk AF populations. Given the substantial clinical impact and healthcare burden associated with AF, the collection of prospective data from local AF cohorts may help establish best practice to reduce AF‐related morbidity and mortality.

## DISCLOSURES

GYHL: Consultant and speaker for BMS/Pfizer, Boehringer Ingelheim, and Daiichi‐Sankyo. No fees are received personally. Other authors: None declared.

## Supporting information

Supplementary Material
